# Book Reviews

**Published:** 1805-10-01

**Authors:** 


### CRITICAL ANALYSIS

OF THE

### RECENT PUBLICATIONS

ON THE

### DIFFERENT BRANCHES OF PHYSIC, SURGERY, AND MEDICAL PHILOSOPHY.

---

*Description and Treatment of Cutaneous Diseases. Order iii. Rashes. Part* 1, *containing the Varieties of Rubeola and Scarlatina. By* ROBERT WILLAN, M. D. F. A. S. London, 1805.

AT length the third part of this valuable work has made its appearance; and though we cannot but regret, in common with our readers, that it has been so long retarded, yet those who know the difficulties of executing such an undertaking with justice, will not accuse the author of delay. The present number being confined to two complaints which are sometimes confounded, the reader will expect the discriminations to be particularly accurate; and in this he will not be disappointed.

The description of Rubeola or Measles, though perhaps more minute from the necessity the author felt of peculiar accuracy, cannot be materially different from our best writers on the subject. However, there are a few facts which are better established than heretofore. The first of these is the period after receiving the infection, when the symptoms commence; and secondly, the period after such commencement, before the eruption shows itself. These, with the varieties to which they are liable, are accurately traced. The period after exposure to the contagion, before the fever commences, or, to use Mr. Hunter's words, the period of the disposition to the disease before its visible action commences, is usually from the sixth to the tenth day. The period of eruption after fever is from the third to the sixth day. Those who are less susceptible may continue for a long time communicating with the sick; but the contagion does not act upon them unless the body be brought into a feverish state by some accidental cause, as, by taking cold, by watching, fatigue, or mental suffering. "I do not recollect," adds the author, "any person wholly unsusceptible of the disease after repeated exposure, as happens in small-pox in a very considerable proportion." But if all are susceptible of the disease, it follows, that all must feel ultimately the effect of the contagion, if exposed to it, even though the body should not be brought into a feverish state by any other cause. May we not therefore be allowed to doubt whether the deranged actions in the constitution from these other causes, may not have produced this protraction of the disease? That such a cause will protract the appearance of the the rash, the author shows by a case, the periods of which he had an opportunity of marking with particular accuracy. He afterwards reduces it to a general law: That, "when a person who carries about him the contagion of the measles, gets a catarrh, or becomes feverish from the causes mentioned above, the contagion begins to operate about the fourth day of such a fever, and then excites fresh paroxysms, which after four days terminate in the eruption. Hence the febrile stage in this instance appears to have the extent of eight days."

Now it is well known that the common period of the acute state of catarrh is about four days. From that time, if the patient is not well, he is at least convalescent, and complains more of the effect of his *cold* than of the disease itself. Is it not therefore probable that the action of measles has been suspended by this new action, that is, the catarrh, after the impression has been received from the contagion; and that when this action from the supervening catarrh has been completed, that of measles commences, and goes through its usual progress? We are led to offer these suggestions to our ingenious author from the following very valuable history.

"I inoculated about the same time three children with the fluid contained in these vesicles, but no effect was produced by the inoculation. A similar trial at the Inoculation Hospital proved more successful. Richard Brookes, aged eighteen, was inoculated by Mr. Wachsell, with fluid from the miliary vesicles in mealses, and with vaccine virus, January 6, 1800. On the 10th there was some redness and elevation of the cuticle at both the inoculated places. 15th. The redness round the part where the lymph of the measles was inserted had disappeared, while the vaccine pock was vivid. 18th. The vaccine disease was over. 22d. He had severe cough, sneezing, and watery eyes, with cold shiverings and fainting. 28th. The measles appeared; his eyes were inflamed, and the lids swollen. 29th. The efflorescence was diffused all over the surface of the body; frequent cough; violent fever. Feb. 1, Efflorescence disappeared; cough and fever much abated. From that time he recovered gradually, and was dismissed in health on the 12th of February."

We have copied this passage not only as an instance of successful inoculation for measles, but also as an illustration of our opinion concerning the causes of the protraction of the disease after the contagion has been received. Here we find the subject susceptible of the two contagions at the same time; and as long as the diseased actions were local, both went on at the same time. But as soon as the constitutional action from vaccination commenced, the local action of measles ceased. When the vaccine process was completed the rubeolous recommenced, and in four days afterwards the cough, sneezing, and other symptoms previous to the eruption came on. In six days more the eruption appeared, and in four more disappeared; making in the whole twenty-six days, a fair allowance for the two diseases. We would not be supposed in this examination examination to question Dr. Willan's accuracy or his abilities, we have fairly stated the question as it appears to us, and leave the decision to our readers.

After a very accurate description of the disease in its most usual progress, the author enumerates some of the anomalous appearances remarked by himself and other writers. We shall transcribe his own, not only on account of their importance, but because they lead to the solution of a difficulty which no one has ventured to touch upon with so much boldness before.

"Some other appearances which occasionally succeed the measles likewise demand attention. These are, 1st. Small hard tumours, like boils, being in the beginning very much inflamed, and sometimes of a livid colour, afterwards suppurating with great pain, and a sanious discharge. They appear mostly on the back, loins, or lower extremities, and are not readily healed. In children there is an analogous eruption of inflamed pustules (Phlyzacia Def. X. 1.) on different parts of the body, but particularly on the feet, legs, thighs, and scrotum.

"2dly. An eruption round the chest, about the mouth, temples, &c. of watery vesicles, in clusters, with an inflamed base, producing much heat, pain, and tingling of the skin.

"3dly. In infants aphthous ulcerations of the tongue and fauces.

"4thly. Soft pustules containing a viscid, straw-coloured fluid, (Achores & Favi, Def. X. 3.) on the head, face, breast, and thighs, succeeded by ulcerations at the corner of the mouth with tumour of the upper lip, sore eyes and ulcerations of the tarsi, discharges from behind the ears, enlargement and tedious suppuration of the submaxillary, occipital, axillary, and inguinal glands, sometimes with pain and swelling of the joints.

"5thly. In some cases where no eruption of pustules, nor superficial ulcerations have preceded, the lymphatic glands of the neck and other parts become considerably enlarged; this appearance is succeeded by a swelling and tension of the abdomen, with hectic fever and emaciation.

"I never saw the rubeola terminate by gangrenous ulcers of the throat, cheeks, gums, &c. or by caries of the jaw-bones, as stated in several respectable authors. Those dreadful symptoms more especially belong to another disease of the present order."

We shall pursue this important enquiry without interruption.

"It may be proper," says our author afterwards. "to notice the 'Putrid Measles,' observed by the late Sir William Watson among the children at the Foundling Hospital, in 1763 and 1768; see Med. Observ. vol. 4. In this disease there was a cough and watery inflamed eyes, but 'the eruption appeared, over nearly the whole body, on the second day;' the fauces were of a deep red colour; the pulse was very quick, but low; the patients complained of extreme weakness, and could not bear bleeding; their oppressed and difficult breathing was attended with great restlessness and anxiety, but with scarce any expectoration throughout; some died under under laborious respiration, more from a dysenteric purging; several were so debilitated that they refused to take almost any nourishment, and sunk quite emaciated, one so late as six weeks after the attack; some cases terminated in mortification of the rectum, pudenda, cheeks, gums, &c. others with caries of the jaw-bones.' These circumstances do not belong to the rubeola, or measles generically considered; they are, indeed, ranked otherwise in Sir W. Watson's own statement respecting the disease, which he refers to the morbilli maligni, or epidemii, described by Morton. Now it must be observed, Dr. Morton expressly maintains that the measles and scarlatina are the same disease, with no more variation in their form than there is between the distinct and confluent small-pox; he has therefore conjoined their principal symptoms (cap. iii.) and wishes to banish the distinction, and the very name of scarlatina from medical language. In this wish Dr. Morton has not succeeded; hence those readers who attend, not to the names of things, but to the things themselves as described, will find that the morbilli maligni, morbilli epidemii, morbilli spurii, and febris morbillosa, pestilentialis, in his writings, have no relation to the measles, but constitute the disease, to which other writers have given the titles of angina maligna, ang. epidemica, ang. pestilentialis, ang. ignea, scarlatina anginosa, scarlatina maligna, &c. &c.

" Sir William Watson probably first adopted Dr. Morton's opinion, and nomenclature, about the year 1768; see Med. Obs. IV. p. 133. In 1763, his technical terms seem to have been different; he says, Med. Observ. p. 136, 'The first person seized with the epidemic measles was on April 21,' but in the weekly Report of the sick to the Hospital Committee, and in the apothecary's book, this case is denominated 'eruptive fever.' The two other cases, said, Med. Obs. p. 137, to have occurred between April 21 and May 4, are not entered in the written books under the denomination of 'measles;' one is termed 'eruptive fever,' the other 'scarlet fever.' From these sources one hundred and eighty children were soon affected with the disease in question, every case of which is termed 'eruptive fever,' no mention being made of measles in the report-book till the latter end of November, when ten cases are entered under the name of 'morbillous fever.' This, however, had no connexion with the preceding 'eruptive fevers,' which, according to the printed account, (p. 137) wholly ceased on June 9.* In

" * Sir W. Watson's statement has led several persons to suspect, that, from the virulent symptoms of a disease alway reputed inflammatory, there must be something amiss in the state of the air, the diet, or general management of the children at the Foundling Hospital. For this suspicion there is no ground. The regulations remain nearly the same as first framed by the Governors, yet their active and intelligent physician, Dr. Stanger, informs me that the measles, during the last twelve years, have never appeared in any other form than as described by Sydenham; and though frequently

In 1766 many of the Foundling children, particularly those placed at Westerham Hospital in Kent, are said to have been affected with 'eruptive fevers and sore throats,' a title not afterwards employed. The measles appeared among the children of the Foundling Hospital at the beginning of March 1770, and continued some time. In May, the scarlatina and measles seem to have occured together, and to be distinguished according to Dr. Morton's nomenclature, as follows,—'Measles,' 'measles and sore throat' or 'measles and ulcerated sore throat,' and measles with 'putrid fever.' The denomination 'scarlet fever and sore throat,' first occurs in the weekly report, Sept. 1, 1787. About the same time, in the prescription book appropriated to the measles, a separate entry is made of scarlatina, this generic title being at length applied, when the disease, after a dreadful ravage during two successive years, had fully impressed the inhabitants of London with a knowledge of its distinctive character, and peculiar virulence."

We shall make no apology for this long quotation on an enquiry leading to so important a discrimination. Dr. Willan's arguments seem almost conclusive, and it is impossible not to admire his industry in following the question so closely. But we cannot help also expressing our surprize when he tells us, that measles have not been fatal at the Foundling beyond the usual proportion; when by the register, it appears, that at two periods taken collectively, about one in thirteen died. Surely, such a mortality as this is beyond the common ratio in this disease. The question is carried much further than our limits will permit us to pursue it, but not further than every diligent enquirer will wish to accompany our author. This part of the work concludes with some short but pointed remarks on the low state of Saracenic or Arabian medicine, even when in its highest reputation.

The next genus in the order of exanthemata or rashes, according to Dr. Willan's arrangement, is Scarlatina. This *genus* is divided into three *varieties*. Scarlatina simplex, anginosa, and maligna, (comprehending that form of the disease in which the efflorescence is confined to the throat.) After a minute description of the most common appearances of scarlatina simplex, our author very accurately points out the peculiar diagnostic marks by which it

---

frequently occurring there, that they have not been fatal beyond the usual proportion. Thus, in the year 1798, twenty-five boys, and forty-four girls, had the measles, six of the latter died. In autumn 1800, twenty-nine boys, thirty-seven girls were affected, and four boys died of the disease, or its consequences. In 1794, twenty-eight had the measles, and all recovered. In 1802, one died out of eight children affected. Particular cases may occur wherein symptoms of putrescence appear during the latter stages; (p. 237). Of such cases I have only noticed five, in a practice of twenty-four years; many practitioners who have been established fifty years, have not seen them in a greater proportion; I never yet conversed with any one who had noted putrid measles occurring epidemically."

it may be distinguished from measles. As this is connected with an enquiry in which we have hitherto followed our author with so much caution, we shall do him the justice to transcribe the passage.

"Although the measles and scarlatina are now known to arise from different modes of contagion, yet so many authors have considered them as varieties of the same disease, that it may be allowable in this place to recapitulate their diagnostic characters.

"1st. The efflorescence in scarlatina generally appears on the second day of fever; in the measles it is seldom visible till the fourth.

"2dly. It is much more full and spreading in the former disease than the latter, and consists of innumerable points and specks under the cuticle, intermixed with minute papulæ, in some cases forming continuous, irregular patches, in others coalescing into an uniform flush over a considerable extent of surface. In the measles the rash is composed of circular dots partly distinct, partly set in small clusters or patches, and a little elevated, so as to give the sensation of roughness when a finger is passed over them. These patches are seldom confluent, but form a number of crescents, or segments of circles, with large intervening portions of cuticle, which retain their usual appearance. The colour of the rash is also different in the two diseases, being a vivid red in the scarlatina, like that of a boiled lobster's shell; but in the measles a dark red, with nearly the hue of a rasberry.

"3dly. During their febrile stage, the measles are distinguished by an obstinate harsh cough, forcing up, in repeated paroxysms, a tough, acrimonious phlegm,—by an inflammation of the eyes and eye-lids, with great sensibility to light,—by an increased discharge from the lachrymal gland, sneezing, &c. The scarlatina is frequently attended with a cough, also with redness of the eyes from an extension of the rash to the tunica albuginea, circumstances which render the distinction between this complaint and measles particularly difficult, if other symptoms be not clear and decisive. On minute observation, however, it will be generally, perhaps always, found, that the cough in scarlatina is short and irritating, without expectoration; that the redness of the eye is not attended with intolerance of light, that the ciliary glands are not affected; and that, although the eyes appear shining and watery, they never overflow.

"4th. Most writers on the subject, in distinguishing scarlatina from measles, and other eruptive complaints, observe that there is a peculiar sensation of anxiety, depression, and faintness, in all cases of it which are attended with fever.

"5th. When the rash appears on the third or fourth day, being scattered, and of a dark shade of colour, as frequently happens in the two latter varieties of scarlatina, the disease may be distinguished from measles by the appearances in the throat, by the rigidity dity of the muscles of the neck, and other peculiar symptoms hereafter to be described."

We have next very accurate descriptions of the two other species of scarlatina, viz. anginosa and maligna. In both these we meet with the caution and minuteness which might be expected from Dr. Willan. Next follow some enquiries, whether the disease can attack a person more than once during life. Amidst the contradictory evidence, the author seems disposed to the negative side of the question, but unwilling to decide. This introduces an historical detail of the different periods at which the disease has been described in different countries throughout the world. The author conceives it was originally introduced among us from the Levant or Mediterranean. It is highly probable that the country first peopled, the most populous and most abounding with the children of poverty, may have been the first among whom a contagious disease was discovered in such full force, and so generally extended as to attract the attention of physicians. The uniformity and fatality of its symptoms would force upon them a conviction that something more was to be described than was met with in Galen and Hippocrates, and that a mode of treatment was to be pursued different from any authorized by these celebrated oracles. With submission however to the great diligence and ingenuity of our author, we cannot help doubting whether the disease described by Aretæus Aetius, and even by Tournefort, can fairly be called scarlatina. No mention is made of a scarlet eruption, and it does not seem unreasonable to impute all the symptoms to the malignant fever with sore throat, generated in the abodes of poverty, especially when we consider how much this disease is increased in proportion to its inciting causes.

In tracing the disease from a later period, Dr. Willan stands on very firm ground. Its milder form he shows satisfactorily to have been described by Ingrassia in Italy, in the beginning of the sixteenth century, and its malignant form by Wierus in Lower Germany, in the years 1654 and 1655. From this period we meet with equal learning and diligence in tracing the disease to our own times. The superficial reader will perhaps fancy this long detail tedious; but the true pathologist, who knows how necessary the greatest accuracy is in ascertaining the true character of every disease, will be aware that nothing which relates to it can be indifferent. He will follow with pleasure his learned instructor in the variety of forms and synonyms under which he detects a disease, sometimes traced back to Hippocrates, and at others considered as a new phenomenon. From this mass of evidence he will with delight perceive the uniformity of nature, even in her deviations from the healthy actions of life, and find new proofs, if any are wanted, of the necessity of uniting in practice a sound judgment, and a bold, if well founded decision, to the most laborious research.

We offer the following extract not only from the interest it has excited in our own mind, but because it contains a kind of summary mary of the arguments that Dr. Fothergill's ulcerous sore throat, as it was at one time called, was only another form of the mild innocuous scarlet fever, which Sydenham describes almost without proposing a method of treatment.

"The 'sore throat attended with ulcers,' which was prevalent in London during the years 1747—8, and of which an accurate account was given by Dr. Fothergill, appears to have been an epidemic scarlatina, like the anginose fever, and ulcerated sore throat described by Huxham. Although many of Dr. Fothergill's cases were certainly of the malignant kind, and he thought proper to insist generally on the putrid tendency of the disease with a view to combat the mode of treatment then employed, yet it is evident from his treatise, that he frequently saw the disease in its mildest form. The rash, of which he has given a full description, coincides exactly with that in the scarlatina. It appeared on the second day of the disease, and began to decline on the fifth. He further observes, that there were 'in general symptoms of recovery on the third, fourth, or fifth day, and that, "some grew easier from the first day of the attack." With respect to the affection of the throat, he says, "The disease terminates in a superficial ulceration of some of the parts about the fauces, with little appearance of sloughs if the disease is very mild; and with large and deep ones, of a white, cineritious, livid, or black colour, if it is more violent." In another place he says, "Where the disorder is mild, a superficial ulcer appears in one or more of these parts, which may easily escape the notice of a person unacquainted with it, as it can scarcely be distinguished from the sound part, but by the inequality of surface." He adds, that under a proper mode of treatment, "it seldom happens, but that the febrile symptoms disappear, the sloughs come off, and the ulcers are disposed to heal in a few days, unless it be where mismanagement at first, malignity of the infection, or an unfavourable constitution, have one or all contributed to increase the disease, and to render its consequences more lasting and mischievous.'

"The disease described by Dr. F. was attended with hæmorrhage from the nose, mouth, and ears, also from the uterus; such discharges of blood take place in every variety of scarlatina, and in the two latter species are often fatal. He has noticed a circumstance peculiarly attributed to the scarlatina anginosa by most writers on that subject; 'Another symptom which requires our attention in the cure of the disease is an excessive faintness, of which they generally complain soon after they are taken ill, and continue to do so, if they are sensible, till the distemper begins to abate.'

"The doctor has elucidated his observations by two instances (p. 52, 56) in which slight cases of scarlatina anginosa, seem to have proved fatal from an imprudent use of bleeding and purgatives. One of the cases so treated, he says, 'was apprehended to be a common scarlet fever.'

"He remarks, in concluding, 'Thus much, however, seems to be true in fact, that in some cases, the disease appears to be of so mild a nature, and so benign, as to require but little assistance from art. Persons even recover from it under the disadvantages of unskilful and injurious management; whilst in others the progress of the symptoms is so rapid, and the tendency to corruption so strong, that nothing seems able to oppose it. Just as it happens in the small-pox; the benign and distinct sort bears ill treatment without injury; in the malignant flux kind, the utmost art and experience are too often insufficient to conduct the distemper to a happy issue. Whether this diversity in the sore throat we are speaking of, is owing to a difference of constitutions, or of seasons, to the different quality or quantity of the contagion, or the manner of receiving it; or whether there are in reality distinct species, may perhaps hereafter be more certainly determined.' *

"That the disease described by Dr. Fothergill was an epidemic scarlatina is further proved by a letter from Dr. Cotton to Dr. Mead, 'On a particular kind of scarlet fever, prevalent at St. Alban's in the year 1748,' published about the same time as Dr. Fothergill's treatise. The disease had undoubtedly spread from London to all the adjacent towns; and Dr. Cotton's narrative affords an opportunity of comparing its appearances in the country, with its form in a crouded metropolis."

A number of writers follow in order, from the descriptions of most of whom it is shown that the three varieties of the disease are from the same source. The most remarkable among these is Dr. Clarke of Newcastle. His description of the worst cases of scarlatina is so exactly similar to Sir William Watson's putrid measles, that it is impossible to doubt they are the same, and his enumeration of the different forms under which it appeared, confirms the three divisions made by our author. Dr. Clarke's table serves as a further proof; and the large opportunities Dr. Binns enjoyed of tracing

---

"* The influence of accidental circumstances on medical practitioners and medical authors was strikingly exemplified in Dr. Fothergill and Dr. Cotton. A popular alarm, first owing to the sudden death of Mr. H. Pelham's two sons, on the same day, by a malignant sore throat, (Gent. Mag. vol. xxvii. Nov. 1739.) and afterwards kept up through reports of the appearance of this supposed pestilential distemper in other parts of the kingdom, occasioned Dr. F's account of it to be read with the utmost avidity. The title and tenor of his publication so far coincided with current opinion, that he soon attained the highest professional eminence. Dr. Cotton's treatise on the same subject had perhaps equal merit with regard to stile and precision; but as he gave an old appellation to a disease certainly not new, his work attracted little attention, and produced him no emolument. The Doctor was, however, consoled by the visitation of his muse, and by the comforts of his rural 'Fire-side.' He declined an invitation to practice in London, considering 'the metropolis as a dangerous and stormy ocean;'—If we can trust the muse, his 'search after happiness,' among calmer scenes, was not ineffectual.

tracing the progress of the disease in the extensive seminary of Ackworth, place the question beyond all doubt.

Having settled this most important point, the author "proceeds to make some observations on the method of treament in the different forms of scarlatina." However pleased we might be with the result of the former enquiry, and with the general uniformity of description discoverable in various writers, making only a moderate allowance for the difference of climates and season, it is impossible to read the present section without some melancholy suggestions on the uncertainty attending our practice in acute diseases. One most important fact seems to meet with general confirmation, that bleeding has been gradually disused, or employed with greater caution. If the subject were less serious, we might smile at the confidence with which other remedies are extolled or condemned by their different advocates and impugners.

Emetics, particularly in the early stage of the disease, seem to meet no opposition. Blisters are a doubtful and uncertain remedy in the opinion of many respectable writers. Our author however does not scruple to recommend them, when the throat is so much swelled as to occasion painful deglutition. He proposes also such a temperature of the chamber as is most agreeable to the patient's feelings, and such as is the common temperature of the season when the disease assumes its mildest form. Very gentle purgatives, administered with caution, are not without respectable advocates; but it is difficult to collect our author's opinion of them, as well as of bark and wine. Probably, he has not found either of them necessary. We shall consider his remarks on acid drinks hereafter. On the subject of gargles, we meet with nothing very new.

In the last division of the disease, *scarlatina maligna*, our author seems to join in the general objection against blisters. No one can dispute the great disposition to gangrene in the worst form of this disease. Bleeding and purging he considers also as constantly hurtful. On this occasion, a just tribute is paid to the late Dr. Fothergill, as "among the first to discourage this destructive practice,"*—in England we presume our author means, for after tracing the practice of blood-letting through Spain and Italy, he adds, "It is extraordinary that after so dreadful a mortality, no physician

* The following note attends this passage.—"Dr. Huxham persevered in blood-letting till the year 1750, though he says, 'It will be constantly found that the pulse as well as the strength sink vastly after the second or third bleeding, and truly very surprisingly after the first.' Dr. Grant was also an advocate for bleeding in angina maligna in 1770," &c.

We should have been glad if our author could have settled a doubt which was often started by the last race of practitioners, viz. whether or no Dr. Leatherland had any share in reforming the practice in this disease. The only printed document that occurs to us on the subject is contained i Chandler's "Treatise on the Disease commonly called a Cold," a pamphlet of which, probably, few have heard more than the title.

sician should have thought of instituting a different mode of practice. None of them, however, would recede from the plan or maxims they had adopted; so that the honour of arresting the progress of this disease, and of effecting its cure, was claimed by a bold empiric, who attracted general attention by advertising a specific remedy, which was merely a cretaceous earth, or bole, said to be the Samian earth recommended by Galen for ulcers of the mouth. We cannot now impute Charamonte's success, which was certainly very great, to an inert powder, but must refer it to collateral circumstances. A principal one seems to have been, that when the Neapolitans and Sicilians were induced to confide in a specific, they no longer submitted to the usual purging and bleedings, which, in opposition to the whole faculty of Naples, Charamonte decidedly reprobated as the causes of the preceding mortality. The event was by no means in favour of medical science at that period; for, soon after the trammels of regular practice were shaken off, we find that the disease became less frequent, and less fatal, though its name impressed terror through many succeeding generations."

By a note, it appears that Charamonte wrote in 1637. Dr. Fothergill's first edition is dated about 1748. We have, as our author observes, no reason to doubt that Huxham continued bleeding till the year 1750. His Dissertation on the Ulcerous Sore Throat is dated 1757. In this he shows how much trouble he had to convince his neighbouring practitioners, "though in general as careful, capable, and judicious as in most parts of England," that the disease was altogether putrid or malignant. Perhaps, had he produced authorities in addition to argument and demonstration, he might have been more successful. When will the professors of a liberal science, and an art altogether practical, direct their whole views to the improvement of that art, or to the encouragement of all the means by which it can be improved? To empirics we owe the bold use of mercury. To empirics, it now appears, we owe the improved method of treating scarlatina maligna. To a female, the introduction of inoculation; and to empirics the improvement of the practice. It is to be admitted that the ignorant boldness of empiricism requires some controul, but it is also entitled to fair enquiry; and it becomes the business of the enlightened practitioner to profit by the errors as well as success of his more hardy antagonist. But it were well if this jealousy were directed only against empiricism; unfortunately we have too many proofs that eminence and usefulness, if they lead to the temple of Fame, conduct us by so steep, so rugged a path, that few arrive there but to leave their ashes. Who is ignorant of the jealousies which Hervey encountered? Who can misunderstand the little querulous hints of Sydenham? Which of us has not witnessed the ignorant petulance which every where attended Hunter? And who can see, with indifference, the constant puny attempts to blast the laurels, or to intwist thorns in the wreath of Jenner.

We

We know it will be urged, that men conscious of their own rectitude and usefulness should view with contempt the malice of their inferiors. This is certainly true. But superior abilities are too often attended with a peculiar delicacy of feeling, which is much increased by the perpetual well-meant adulation which by degrees becomes habitual to them. We must therefore consider these inconveniencies as inseparable from the nature of man, and only rejoice that as civilization improves, a sense of decency restrains most of us from the expression of passions which are held in universal abhorrence, and which recoil with tenfold severity on those who indulge them. But to return to our subject.

The next remedy suggested by Dr. Willan, on the authority of the best practitioners and his own experience, is emetics. Of these he speaks in very favourable terms. The subject of gargles is fairly and amply discussed. Tepid bathing and cold affusions are next considered. Again, our author shows the same caution in recommending the exhibition of bark and wine, but with his usual candour produces all the evidence on each side. For some time past, acids have been considered as the principal remedy in this disease. The number of authorities quoted in their favour exceeds those which have recommended any other remedy excepting bark, against the admission of which, at some periods, many objections occur. In scarlatina anginosa, *acids* seem the only remedy after emetics, on which our author relies during the early stage of the disease; and for this same disease Dr. Withering recommends *alkalies*. We do not find any objection made to *acids* in scarlatina maligna; the bark and other remedies are said to be necessary in other stages and circumstances of the disease: and here again we have a quotation from an author, who describes "universal efflorescence, extremities swelled, fœtid ulcers, particularly about the parts of generation, throat swelled, inflamed, often ulcerated, and respiration almost prevented, scorching fever, raging delirium, situation horribly alarming; yet, continues this fortunate practitioner, in all these variations of the disease, the *volatile alkali* was my specific, which I administered to between two and three hundred patients successively and successfully!" *Who shall decide*, &c. However, as Dr. Withering recommends alkalis on account of their diuretic quality, and as acids have this property in common with them, why should we not insist on a greater attention to the *urinary* secretion? Huxham remarks, that the "urine was often in small quantities;" and in another place, that "if the animal salts are not duly and constantly carried off by urine, they are highly destructive." It is true, he expresses many objections to the use of alakaline salts in this disease, yet this might be owing to the imperfect knowledge of chemistry in those days. But perhaps we shall be told that it is more the business of reviewers to attend to the suggestions of others than to offer their own.

The rest of this number is occupied in considerations concerning a preservative from the contagion of scarlatina for persons within its its influence, and still more concerning the means of preventing the spreading the disease whenever it is found to exist. On this last point we find our author, as usual, very full of useful information. A valuable history is given of the progress of scarlatina in the school at Ackworth, of the inutility of all preservative means from fumigations, or any thing less than a most careful separation. It is highly probable that the economy of this school, like whatever is managed by this class of the English public, is peculiarly exact as to cleanliness and good order. If this is the case, though such a caution may prove the means of keeping out infection, and a complete preservative against typhus fever, it will also render the inhabitants of the seminary more susceptible of effects from a most slightly tainted atmosphere, inasmuch as they are accustomed only to breathe a purer. That such consequences follow from such causes all analogy teaches us. The impropriety of sending children from school, who are already infected, is warmly insisted on, and Dr. Blackburn's valuable remarks on that subject are properly enforced. Several quotations as well as observations of our author show the progress of infection in certain instances, and the manner in which it might and ought to have been prevented. Lastly, a few of the clauses concerning quarantine, are quoted, to show that the proposed restrictions are less oppressive than laws which have long since been sanctioned and sumbitted to without murmur.

Such is this performance which it is unnecessary we should recommend to our readers. It must find its way into every library, should be carefully perused by every practitioner, and must become a standard authority in ascertaining the true character of the diseases it undertakes to describe. We wait with anxiety but not with impatience for the succeeding numbers, well aware how arduous the undertaking is, and how anxious the author must be to do justice to the reputation he has already acquired. Aware likewise of the readiness with which true merit meets reproof, we take the freedom to hope, that on future occasions we shall see more of the author and less of his authorities. Nothing can be more unsatisfactory than to read accounts of never failing remedies, implying indiscriminate censure on every other practice. Pleased as we may feel with the candour in which our author recapitulates what has been done by his predecessors, we should gladly have admitted more decision from one who has enjoyed so many advantages, and improved them so well.

---

*An Epitome of Infantile Diseases, with their Causes, Symptoms, and Method of Cure; translated from the Latin of Dr. Heberden, by* J. Smyth, M. B. *of Matlock, Uttoxeter.*

This work consists, as its title imports, of a summary view of Infantile diseases. It is an avowed translation from the Latin pamphlet lately published on the same subject by Dr. Heberden, and the translator's object is to diffuse the benefits, which he himself derived derived from the original publication, through a wider circle, and to furnish parents in particular with the fund of practical knowledge which it contains. These, it will be allowed, are motives strong enough to justify the author's undertaking, and the work itself it is presumed will be found a useful compendium of the kind, and worthy of the notice and attention of such persons as may be entrusted with the health of infants. Though the work scarcely extends through eighty pages, there are no fewer than sixty-three chapters, each chapter containing a succinct account of the cause, symptoms, and approved treatment of one particular disease. Three new chapters, viz. the forty-fifth, forty-sixth, and sixty-third, on the nettle-rash, the state of the pulse, and the fistula lachrymalis, are added, and also several notes, chiefly containing results of the translator's practice, together with a copious extract from the works of Dr. Jenner, relating to the cow-pox. Upon the whole, however, this epitome is better calculated for the use and reference of professional students than of common readers, and it may surely be reasonably questioned whether parents in general, or any who have not made the profession their study, are qualified to undertake the management of diseases so superficially noticed as they necessarily are, within these narrow limits; indeed it is evident, had the original work been designed for popular use, it would have been conducted on a very different plan.

As far as the translation goes, there is no doubt but it is faithful; the language is correct and intelligible; extremely literal; and in professional treatises, what would we have more? But when Dr. S. speaks for himself, as he does in his dedication and preface, he cannot be said to have wielded the pen, as it is not to be questioned he wields the pestle, like an experienced general. In the dedication, while he is urging his motives with proper and due solemnity to the President and Fellows of the Royal College, he bursts forth into this sublime and unaccountable phraseology: "*When philanthropy instigates and duty commands, nothing less than torpid apathy can resist the inspiration.*" This is very fine when separated from the context, but it is absolutely lost in the blaze of the concluding sentence: *This stimulating and coercive impulse forcibly urged the propriety of the present undertaking.*" This passage, we may venture to assert, approaches as near as possible to the perfection of the "Bathos or Profund."

In the preface Dr. S. mentions the occurrence of circumstances, which prevented his giving this publication a full share of his attention, and therefore apologizes for trifling inaccuracies; but to this it might be replied, that subjects of this nature, involving the health and happiness of so many interesting claimants upon our sympathy and compassion, ought to receive full consideration, before they are given to the public, or not to be published at all, and that even trifling inaccuracies may justly be deemed inexcusable when the consequences might be both dangerous and fatal; but in truth on a careful perusal, no such inaccuracies appear in the the present work; and this avowal must therefore be attributed not to any undue neglect on the part of the author, but rather to his excessive diffidence, and his care to avoid not only the reality, but even the appearance of error.

---

*A Letter occasioned by the many Failures of Cow-pox. From* John Birch, Esq. *Surgeon to His Royal Highness the Prince of Wales, &c. addressed to* W. R. Rogers, *Author of the Examination of the Evidence before the House of Commons, &c.*

Mr. Birch commences with recommending to Mr. Rogers to reprint his pamphlet. Is Mr. Birch so little of an author as to doubt a brother's readiness to get up a new edition whenever the first is sold off? He cannot surely wish his friend and pupil merely to reprint the title with a view of bringing his (Mr. Birch's) name into the page. However, if the two are tacked together, we recommend to the Royal College of Surgeons to circulate them as much as possible. A few extracts will explain our meaning. In the first page,

"Inoculation (says Mr. B.) has hitherto been considered as distinctly the province of the *surgeons;* the success of it, and the alleviation of *its* distressing symptoms (aye, Mr. B. and of *our* distressing symptoms, the College should add) depend on SURGICAL assistance. *It is a melancholy consideration* therefore to think, that this branch of practice should be taken from those who alone ought to exercise it, and transferred to persons, some of whom are totally ignorant of our profession."

Mr. B. further remarks, "It would have been more regular and more to the interests of society, as the experiment was *surgical,* to have consulted the *College of Surgeons,* and to have collected their approbation before a parliamentary reward was adjudged."

"But (continues our author) the object of the projectors of vaccination, was, I fear, not so much the desire of doing general good as that of securing to themselves and to midwives, if the experiment should succeed, the absolute command of the nurseries to the entire exclusion of *surgeons.*"

Proh pudor! oh shame and equal folly, thus to simplify a profitable branch of practice, and commit the charge of inoculating a whole nursery to women! and all out of spite to the surgeons. We shall offer only one extract more to convince our readers, that we are not afraid of calumny, even when it comes from Mr. Birch.

"A monthly Medical Journal, which has spread the mischief of vaccination widely, and *which till the last month has been shut against every statement which could affect its credit,* now acknowledges failure upon failure, attested by one practitioner after another."

It is probable Mr. Birch's letter, though we believe it is circulated gratis, may be less read than our account of it. We are therefore glad to give his charges against us this publicity, as our readers can judge on what foundation they are supported. If indeed deed, Mr. Birch writes for the nurseries, which he is so anxious to reclaim, he may probably succeed; as those foolish old women do, who alarm children into good behaviour by stories of hog-goblins. Indeed, from the general tenor of the letter, and particularly from the boldness of the last quotation, we should have considered the whole rather directed to nurses than to the gentleman whose address it bears.

---

*Answers to all the Objections hitherto made against Cow-Pox*; *by* Joseph Adams, M. D. *Physician to the Small-Pox and Inoculation Hospitals, and Author of "Observations on Morbid Poisons, &c.* London, published for the Benefit of the Small-Pox Hospital.

This little Tract being intended for the public at large as well as the profession, the author has carefully avoided all technical terms. After an exordium, in which he expresses his surprise, that the objections to vaccinate should be so frequent in England, though unknown in every other part of the world; he comprehends these objections under three general heads.

1st, That the cow-pox is no security against the small-pox.

2dly, That it is only a security for a time; and,

3dly, That it introduces other humours into the constitution.

The first is considered as so completely answered by every concurring fact, as to be now given up by all reasonable people.

That it is only a security for a time is admitted to be urged by a very well intentioned author. But even this Gentleman, it is added, is now "led to believe that vaccination in the hand may be a security for life, though in the arm only for a time." However, the Doctor admits that small-pox may occur after vaccination, without any impeachment of the practice, in one of the three following ways.

1st, By imperfect vaccination.

2dly, By the constitution being under the influence of some other disease at the time of vaccination; and,

Lastly, By the person being liable to the small-pox twice.

The first can only be detected by the most accurate examination of the pustule during its whole progress by a person well acquainted with the appearances to be expected.

For the second, the author refers to those papers published by Dr. Jenner and himself in our Journal.

That the same person may have small-pox twice, he proves by well authenticated cases, resting on unexceptional authority, and recorded before vaccination was introduced. These he shows were sufficient to convince him, when he published his Morbid Poisons in the year 1795, not only that small-pox might occur a second time, but also that a certain period would be necessary before the susceptibility to the disease should return.

Respecting the last charge of the inoculation of humours from the cow, Dr. Adams remarks, that it would be absurd to suppose no no children had breakings out after vaccination, though it will be found, that by it, many more have been cured of their eruptions. But cutaneous diseases following cow-pox have been always temporary, and ceased without any remedies; whereas, those which have followed small-pox have been much more serious, and often fatal. This he imputes to the greater violence of the disease in small-pox, and not to any effects from inoculation. To prove this, he shows, that the most dreadful consequences have occurred from the natural small-pox. But, adds our author, whence can these humours proceed, which are imputed to vaccination? Is it from the cow, or the person from whom the matter is taken? If from the latter, we are not likely to fare better under small-pox inoculation? If from the cow, let us, at least, recollect who are the subjects in whom the cow-pox was first discovered, and who receive it, though with less violence, sometimes, two or three times in their life. These are the plump English dairy-maids! celebrated through the world for their rosy cheeks, their sleek arms, and stout constitutions. This, he adds, may be enough to do away all the nonsense which has been promulgated about the danger of inoculating humours from an animal, whose milk makes the principal part of our children's food; whose flesh is the source of old English courage, and whose breath is not only fragrant, but salubrious.

Such are the arguments contained in this pamphlet, which we have endeavoured to compress as much as possible. Those who wish to see them more at large must consult the Tract itself, which concludes with a general advice on the score of our duty to others; that we should no longer continue to inoculate a disease, which may spread by effluvia, when we may not only secure our children without danger to others, but with no hazard and little inconvenience to themselves.

An Appendix is added, giving several well authenticated instances of small-pox after inoculation for that disease.

